# Supplementary material for: 3-O Sulfated Heparan Sulfate (G2) Peptide Ligand Impairs the Infectivity of Chlamydia muridarum
Source: Biomolecules. 2025 Jul 12;15(7):999. doi: 10.3390/biom15070999 (PMC12292407; doi:10.3390/biom15070999)
Supplement: Supplementary file 1 [file biomolecules-15-00999-s001.zip › biomolecules-3631125-supplementary.pdf]

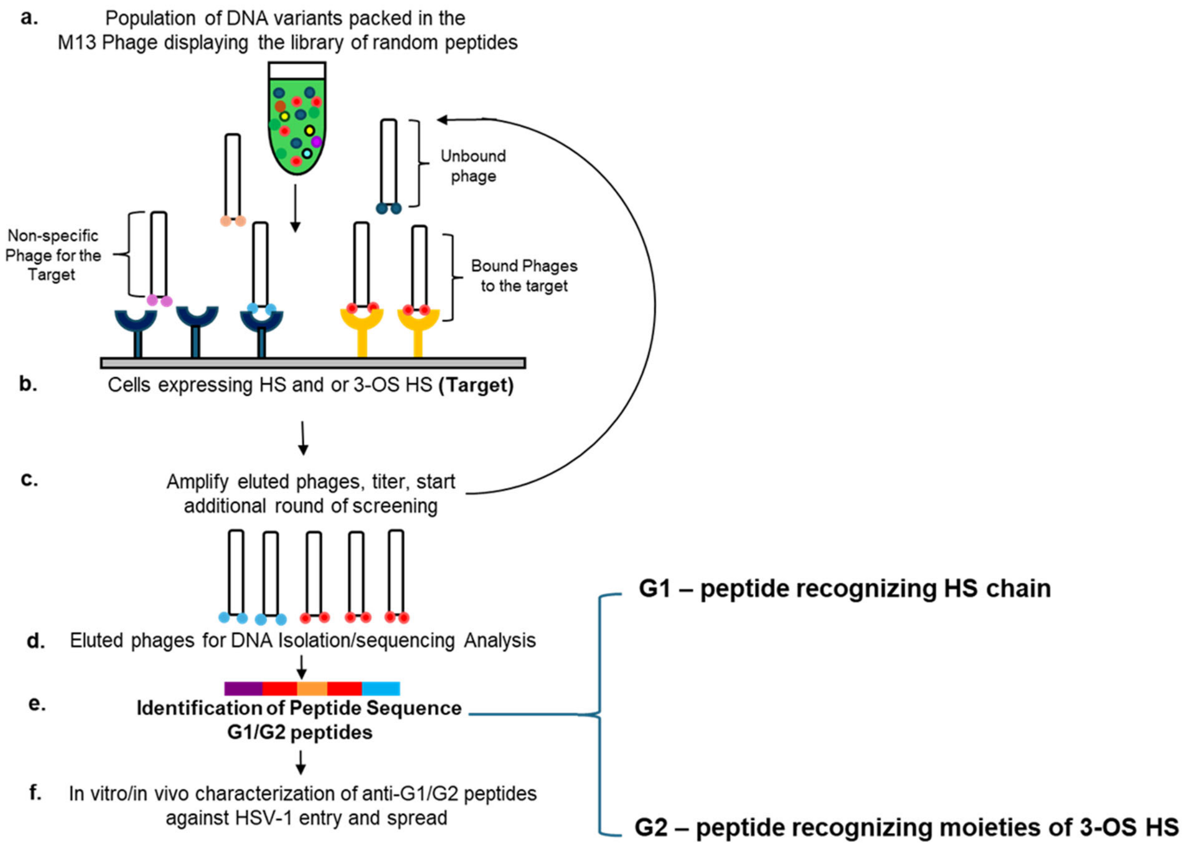

Supplementary Figure S1. Graphic depicting the steps (a-f) involved during phage display library screening of M13 phage against heparan sulfate (HS) and 3-O sulfated heparan sulfate (3-OS HS) to isolate and characterize G1 and G2 peptides.

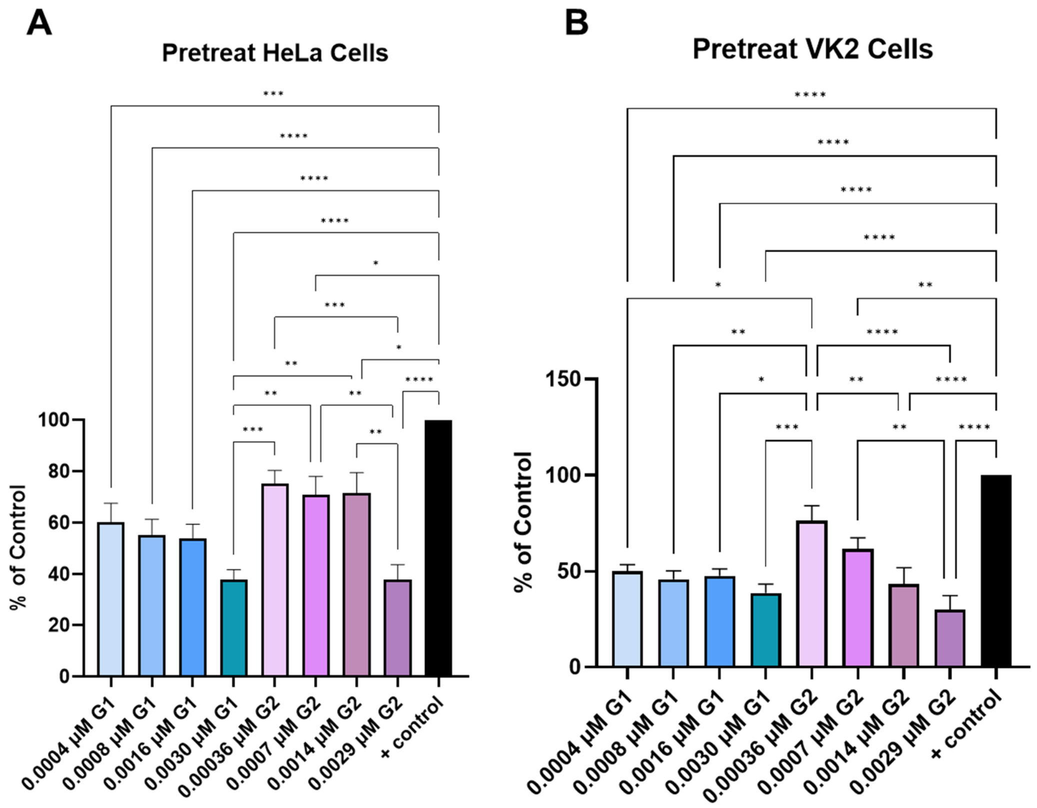

Supplementary Fig. 2

Supplementary Figure S2. Effect of the pretreatment with various concentrations of G1 and G2 peptides on HeLa cells (panel A) and VK2 (panel B) followed by infection with *C. muridarum* as determined by fluorescent microscopy. Inclusions were stained and representative images were shown. The number of inclusions were counted and the percentage of infected cells treated with G1 or G2 compared to non-treated infected cells only (positive control) was calculated (Mean ± SEM, *$p \leq 0.05$, **$p < 0.005$, ***$p < 0.0005$, and ****$p < 0.0001$). Data is a representative of the results of three independent experiments (n=3) performed in triplicates.

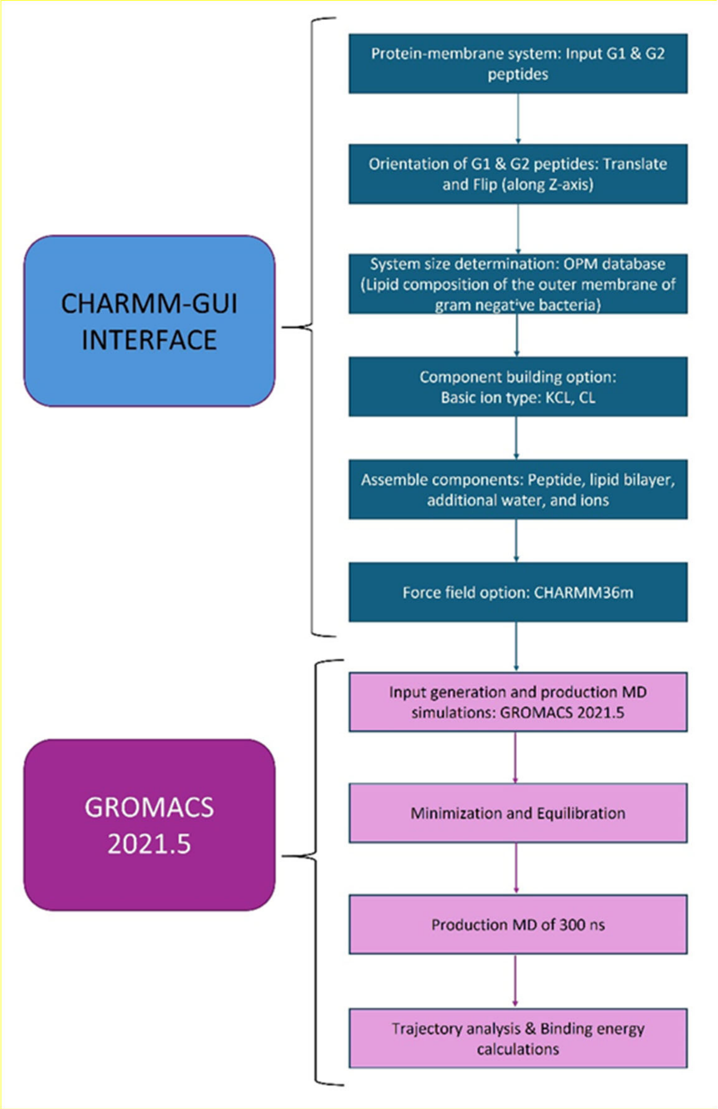

Supplementary Figure S3. The workflow representation of *Chlamydia muridarum* lipid bilayer building and simulation protocol.
